# Examining the influence of turbulence on viscosity measurements of molten germanium under reduced gravity

**DOI:** 10.1038/s41526-022-00238-z

**Published:** 2022-11-24

**Authors:** G. P. Bracker, Y. Luo, B. Damaschke, K. Samwer, R. W. Hyers

**Affiliations:** 1 Department of Mechanical and Industrial Engineering, University of Massachusetts, Amherst, MA USA; 2 Institute for Materials Physics in Space, Deutsches Zentrum für Luft- und Raumfahrt, Köln, Germany; 3 I. Physikalisches Institut, Georg-August-Universität, D-37077 Göttingen, Germany; 4 Department of Mechanical and Materials Engineering, Worcester Polytechnic Institute, Worcester, MA USA

**Keywords:** Theory and computation, Characterization and analytical techniques, Fluid dynamics

## Abstract

The thermophysical properties of liquid germanium were recently measured both in parabolic flight experiments and on the ISS in the ISS-EML facility. The viscosity measurements differed between the reduced gravity experiments and the literature values. Since the oscillating drop method has been widely used in EML, further exploration into this phenomenon was of interest. Models of the magnetohydrodynamic flow indicated that turbulence was present during the measurement in the ISS-EML facility, which accounts for the observed difference.

Containerless processing techniques, like electromagnetic levitation (EML), allow strongly reactive materials to be studied at high temperatures at which the material would both melt and react with its container. During electromagnetic levitation, the sample is supported by an electromagnetic levitation field and contained by the surface tension of the melt^1^. By doing so, the available heterogeneous nucleation sites are reduced and the undercooled region of the melt is available for study, extending the range of temperatures accessible for measurement. Recent experiments on the International Space Station (ISS) in the Electromagnetic Levitation (EML) facility and in parabolic flight experiments have taken measurements on the density, thermal expansion, viscosity, and surface tension of molten germanium^2,3^. An extensive and accurate understanding of the behavior of these thermophysical properties is necessary both to improve our understanding of the fundamental nature of molten semiconductors and to facilitate efficient manufacture using such materials. The semiconductor industry is interested in the thermophysical properties of germanium and Si_1-x_-Ge_x_ alloys that would allow the band gap to be precisely tuned for various electronic applications^4–6^. The results of these viscosity measurements are shown in Fig. 1, where it can be seen that the viscosity measurements taken during the parabolic flight experiments on pure germanium are approximately an order of magnitude larger than the measurement taken in the ISS-EML facility^3,7^. The viscosity measurement was taken at 1310 °C and observed to be 2.9 mPa s^7^ in the ISS-EML. However, ground-based measurements by Gruner^8^, using an oscillating cup viscometer, indicate a viscosity of 0.367 mPa s, an order of magnitude lower. Gruner’s oscillating cup measurements were fit to the Arrhenius relationship, given in Eq. (1), for pure germanium in which η_∞_ = 0.206 mPa s and E_η_ = 7.60 kJ/mol:

1 $$\eta \left( T \right) = \eta _\infty \ast \exp \left( {\frac{{E_\eta }}{{RT}}} \right)$$

**Fig. 1:**
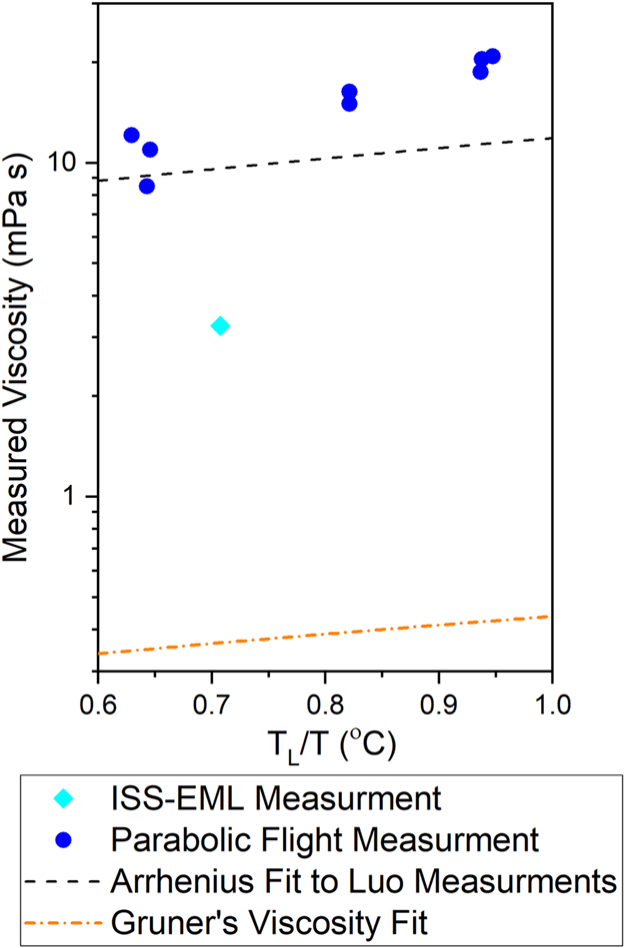
Viscosities measured during parabolic flight testing across a range of different temperatures and compositions of Ge-Si adapted from ref. ^2^. In light blue, the measurement taken in the ISS-EML is plotted^3,7^. The result from the ISS-EML is about an order of magnitude lower than the results of the parabolic flight experiments. However, ground-based techniques are still an order of magnitude lower^8^.

In the microgravity experiments during parabolic flight^1^ and in the ISS-EML^3,7^, the oscillating drop method was used to measure the surface tension and the viscosity of the melt over a range of processing temperatures in the facility described by Lohöfer^9^. The oscillating drop method utilizes the electromagnetic force field to excite surface oscillations in the sample. The properties of the melt are inferred from the response of the oscillations according to the relationships calculated by Rayleigh^10^ and Lamb^11^. The frequency of the oscillations is determined by the surface tension and the damping coefficient is determined by the viscosity. Lamb’s equation relating the damping coefficient to the viscosity of the melt assumes that there is no flow other than the flow driven by the surface oscillations and that that flow is laminar. While it has been assumed that laminar flow driven by the EML forces can be superimposed over the flow driven by the perturbations without affecting the surface oscillations^12,13^, turbulent eddies greatly accelerate the damping. During turbulent flow, the momentum of the surface oscillations is redistributed by the turbulent eddies and damping is dominated by the turbulent dissipation rather than by the inherent viscosity of the liquid. As a result, it is important to calculate the Reynolds number describing the flow within the drop^14,15^.

However, it is difficult to observe the behavior and velocity of the flow during EML experiments directly. In the liquid state, germanium is a metallic conductor. Like other molten metals, germanium is opaque, preventing optical access to the internal flow. While surface particles may be present in EML experiments, these particles are swept into the stagnation lines of the flow and do not provide quantitative insight into the flow behavior. Instead, magnetohydrodynamic models are used to relate the experimental conditions and properties of the melt with the resulting internal flow of the sample.

The flow was modeled using conditions present in the ISS-EML experiment at 1310 °C when the property measurements were taken. The magnetic model used 1.52 × 10^6^ S/m for the conductivity of molten germanium as measured by Skinner at 1250 K with negligible changes as a function of temperature over the range of interest^16^. The EML force field is defined by a control voltage of 7.72 V for the positioner and a control voltage of 0.00 V for the heater circuit, which was ON at the time of interest. At this temperature, the density was calculated to be 5308 kg/m^3^ using Iida and Gutherie’s density fit^17^ and the viscosity was calculated to be 0.367 mPa s using Gruner’s viscosity fit^8^. The resulting maximum flow velocity in the drop was then calculated to be 0.147 m/s, corresponding to a Reynolds number of ~17,000, which indicates clearly turbulent flow. The flow vector field and turbulent viscosity are plotted in Fig. 2. During the measurement, the EML force field was dominated by the quadruple positioner field in which the largest forces are applied around ±45° from the equator of the sample while also exponentially decreasing toward the sample interior. In this system, the maximum flow occurs near the surface of the sample, while the center region of the sample has the larger turbulent viscosity. However, other distributions of turbulence have been calculated for other systems, such as those discussed in refs. ^18–20^. Despite these differences in the distribution of turbulence within the sample, accelerated damping would be present in all these cases in which turbulence is present. Further investigations are necessary to better understand the behavior and distribution of turbulent eddies in EML flows.

**Fig. 2:**
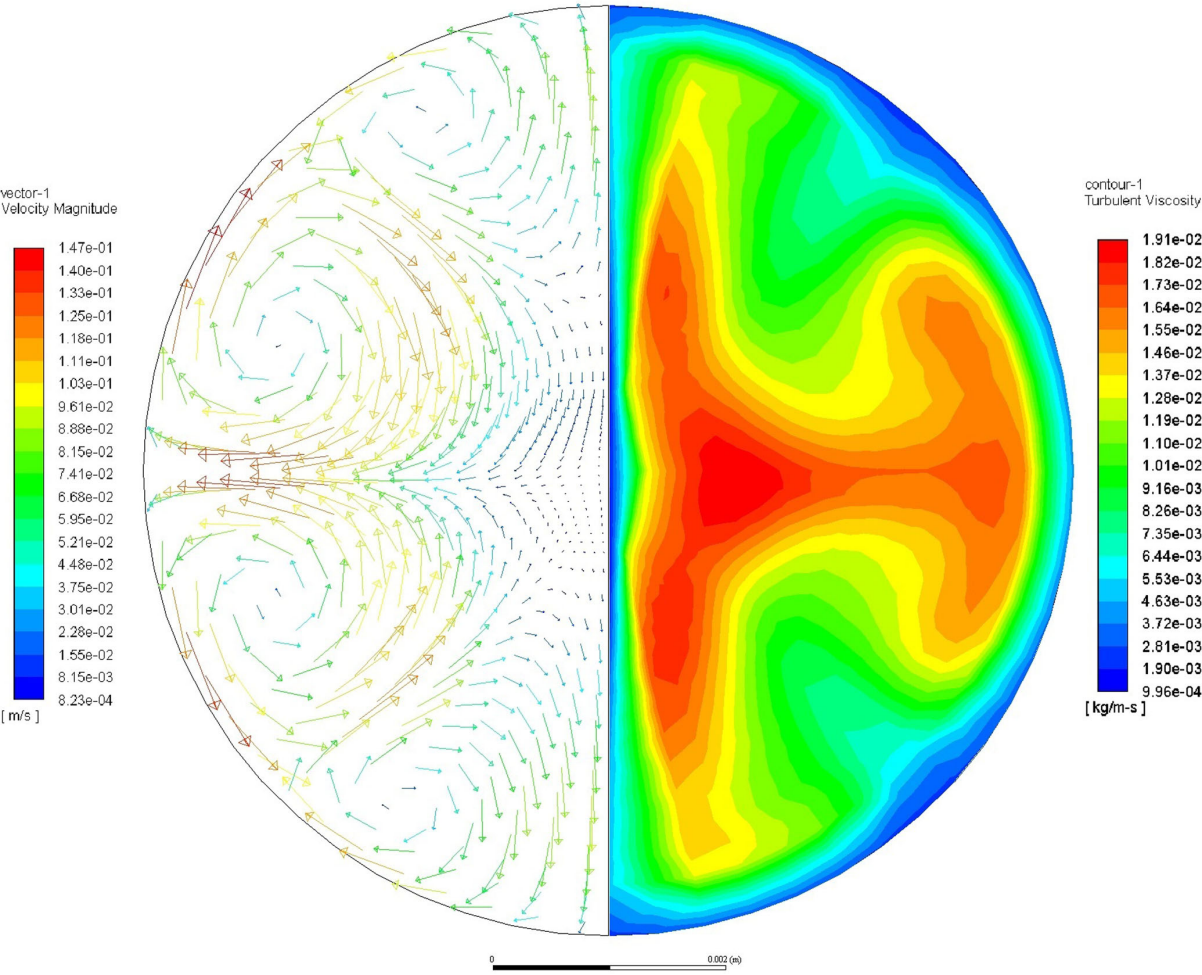
The internal flow within the molten germanium sample at the time of the ISS- ML measurement. The velocity vectors are plotted on the left-hand side in which the flow is driven into the sample at ±45° from the equator of the sample. The turbulent viscosity contours are plotted on the right-hand side, which provides a measure of the kinetic energy is dissipated by the turbulent eddies.

Under these conditions, the assumptions of Lamb’s equation are not satisfied. The model shows that the damping reported by Luo et al.^3,7^ from the ISS-EML experiments was largely due to turbulent dissipation. The higher viscosity values reported by Luo et al.^7^ on the parabolic flight experiments correspond to even higher flow velocities than the models presented here. These higher flow velocities explain the even faster damping observed for the parabolic flight experiments.

The flow within the drop was explored over a wider range of conditions to find whether or not laminar flow was accessible for any combination of parameters. The flow was modeled using the EML force fields used during the experiment over the range of cooling. The slowest flow occurred at recalescence, immediately before solidification. Recalescence, in this sample, occurred when the sample was at 885 °C. At this time, the control voltages on the EML field were 3.90 V positioner and 0.00 V heater, with the heater circuit ON. The density at 885 °C was calculated to be 5516 kg/m^3^ using Iida’s density fit^17^ and the viscosity was calculated to be 0.454 mPa s at 885 °C using Gruner’s viscosity fit^8^. The resulting maximum flow velocity in the drop was then calculated to be 0.0744 m/s which corresponds to a Reynolds number of 7240, which still is more than an order of magnitude larger than the expected laminar-turbulent transition at Reynolds number 600^21^.

In addition to the turbulent positioner-induced flow, the excitation pulse drives rapid acceleration within the sample driving even faster flows. Work by Xiao has shown that pulse-turbulence may increase the apparent viscosity by 2–8 times^22^. Following the excitation pulse, the flow slows by the viscous dissipation of momentum. In EML experiments, the length scale over which viscous dissipation of momentum takes place has been shown to be the radius of the recirculation loops. In heater-dominated flows, like those driven by the excitation pulse, this has been shown to be about 1/3.5 times the radius of the sample^23^. For a sample of this size and the viscosity taken from Gruner^8^, the turbulent viscosity in this sample at the end of the heating phase is 62.12 Pa s. The effective viscosity of the system includes both the turbulent viscosity and the viscosity of the melt, 0.367 mPa s; therefore, the effective viscosity is dominated by the effects of turbulent flow. The accelerated flow due to the excitation pulse would require about 0.11 s to dissipate however, the timescale for viscous dissipation of momentum by the viscosity of the melt alone, as occurs in laminar flows, is about 230 s. During oscillating drop experiments on the ISS, the damping coefficient can be calculated to estimate the damping for the oscillations; in a sample of these properties, the damping coefficient is 46.3 s. The damping in the experiments on the ISS corresponds to positioner-dominated flow after 0.11 s following the excitation pulse. During the measurement, the observed damping corresponds to both the viscosity of the melt and the turbulent viscosity driven by the positioner-dominated flow.

The turbulent flow in the sample was further validated through a video of the experiment on the ISS in which oxide rafts on the surface of the sample can be seen to move chaotically throughout the cycle up to recalescence. The chaotic motions indicate that the flow was, in fact, turbulent. The turbulent flow at the minimum flow conditions indicates that it is not possible to achieve laminar flow in EML for a sample of this size with such low viscosity.

Measurement of the viscosity of molten germanium using oscillating drop in microgravity EML on the ISS^3^ and in parabolic flights^2^ reported values much higher than those obtained using an oscillating cup viscosimeter^8^. Models of fluid flow in the EML samples reveal that the reported difference in viscosity was caused by turbulent flow in the levitated samples. This turbulence is not always observed in EML, but only for specific combinations of sample size, material, and operating parameters. Further calculations show that for germanium samples of this size, the turbulence persists for all achievable experimental conditions. It is recommended that the flow effects are characterized using projected experimental parameters with the properties of the melt during the planning phase to ensure that the experimental conditions satisfy the requirements of the measurements during the experiment.

## Methods

### Computational fluid dynamics models

Since the flow of the drop cannot be directly observed during most EML experiments, models are used to assess and quantify the flow behavior. The flow is driven by the EML force field, which is calculated using the coil geometry, sample geometry, conductivity of the melt, and the applied current to the EML system. Further details on the magnetic model are described by Hyers et al.^1^ and Bracker et al.^24^ The flow is modeled using computational fluid dynamics (CFD) in conjunction with the magnetic model. The work presented here uses ANSYS Fluent to calculate the flow present during the experiment.

The CFD model for the microgravity EML experiments is defined by the following boundary conditions: The free surface of the drop cannot be crossed by the flow and is free of traction. Second, the sample is represented by a two-dimensional axisymmetric mesh. At the axis of symmetry, the derivatives must be zero.

The model has been validated against a physical experimental case in which the sample, a copper-cobalt alloy, formed a two-phase liquid^25^. This case provided a rare opportunity to use particle imaging velocimetry to directly quantify the flow on the surface of the drop. This work by J. Lee et al. found that the model agreed with the experimental accuracy, better than 7% error^24^.

The flow was analyzed using both laminar and turbulent flow models. The laminar model directly solves the discussed Navier-stokes equations in ANSYS Fluent. Prior work has found that the laminar-turbulent transition occurs near Reynolds number 600, with flow above this observed to be turbulent while flow described by lower Reynolds numbers is seen to be laminar^21^. Microgravity EML experiments can display both laminar and turbulent flows. In EML, the Reynolds numbers of the turbulent flow are relatively low, when compared to traditional turbulence studies. Despite the low Reynolds numbers, turbulent EML flow maintains the key characteristics of turbulence: chaos, mixing and vorticity. Turbulent flow in EML is best described by the RNG K-ε turbulence model^26^, which includes additional transport equations to the Navier-Stokes equations to account for turbulent kinetic energy and dissipation, and account for low Reynolds number effects^27,28^.

### Reporting summary

Further information on research design is available in the Nature Research Reporting Summary linked to this article.

## Supplementary information

Reporting Summary Checklist

## Data Availability

The data generated and analyzed during the current study are available from the corresponding author upon reasonable request.
