# Publisher Correction: Examining the influence of turbulence on viscosity measurements of molten germanium under reduced gravity

**DOI:** 10.1038/s41526-023-00264-5

**Published:** 2023-02-20

**Authors:** G. P. Bracker, Y. Luo, B. Damaschke, K. Samwer, R. W. Hyers

**Affiliations:** 1 Department of Mechanical and Industrial Engineering, University of Massachusetts, Amherst, MA USA; 2 Institute for Materials Physics in Space, Deutsches Zentrum für Luft- und Raumfahrt, Köln, Germany; 3 I. Physikalisches Institut, Georg-August-Universität, D-37077 Göttingen, Germany; 4 Department of Mechanical and Materials Engineering, Worcester Polytechnic Institute, Worcester, MA USA

**Keywords:** Theory and computation, Characterization and analytical techniques, Fluid dynamics

Correction to: *npj Microgravity* 10.1038/s41526-022-00238-z, published online 24 November 2022

In the original version of the Article, Figures 1 and 2 were inadvertently interchanged during typesetting and publication whereas the legends were correctly placed.

This has been corrected in both the PDF and HTML versions of the Article.

